# Supplementary material for: Transcriptomic Insights into Mechanisms of Early Seed Maturation in the Garden Pea (Pisum sativum L.)
Source: Cells. 2020 Mar 23;9(3):779. doi: 10.3390/cells9030779 (PMC7140803; doi:10.3390/cells9030779)

Zhewan−1(10)

Global Summary

%DE = 0.84

genes with fdr < 0.2 = 1742 (537 + / 1205 −)

genes with fdr < 0.1 = 1610 (464 + / 1146 −)

genes with fdr < 0.05 = 1489 (391 + / 1098 −)

genes with fdr < 0.01 = 1129 (223 + / 906 −)

genes in genesets = 0

<FC> = 0

<t−score> = −42824.62

<p−value> = 0

<fdr> = 0.16

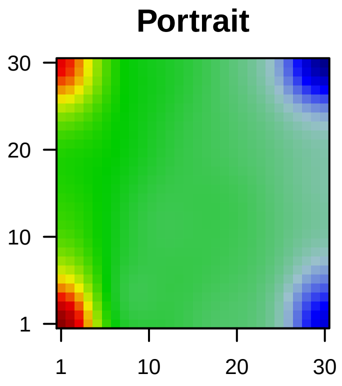

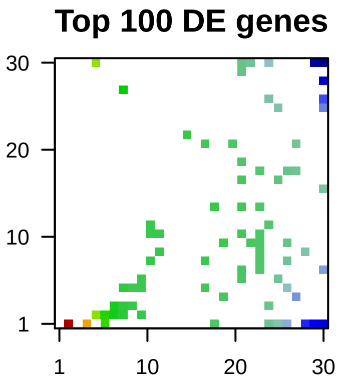

Global Genelist

Rank	ID	log(FC)	p−value	fdr	Metagene	Description
1	comp39208_c	−0.03	0e+00	3e−13	23 x 14	
2	comp17322_c	−0.07	9e−16	1e−12	18 x 1	
3	comp19514_c	−0.04	4e−15	3e−12	23 x 18	
4	TRINITY_DN5	−1.68	1e−14	9e−12	30 x 1	
5	comp20228_c	−0.03	4e−14	3e−11	10 x 5	
6	TRINITY_DN2	−0.02	1e−13	3e−11	17 x 21	
7	comp22509_c	−0.06	2e−13	3e−11	8 x 2	
8	TRINITY_DN2	−1.92	3e−13	3e−11	30 x 30	
9	TRINITY_DN4	−0.46	4e−13	3e−11	29 x 30	
10	TRINITY_DN3	−0.09	4e−13	4e−11	30 x 16	
11	TRINITY_DN7	−0.48	5e−13	6e−11	30 x 1	
12	TRINITY_DN2	−0.09	7e−13	6e−11	21 x 30	
13	TRINITY_DN1	−0.33	1e−12	6e−11	30 x 1	
14	TRINITY_DN3	−0.19	1e−12	6e−11	7 x 3	
15	TRINITY_DN1	−0.33	1e−12	6e−11	30 x 28	
16	TRINITY_DN3	−0.34	1e−12	8e−11	27 x 4	
17	TRINITY_DN1	−0.19	2e−12	8e−11	29 x 1	
18	TRINITY_DN1	−0.13	2e−12	1e−10	7 x 2	
19	TRINITY_DN1	−0.21	2e−12	1e−10	30 x 1	
20	TRINITY_DN3	−0.62	3e−12	1e−10	28 x 1	

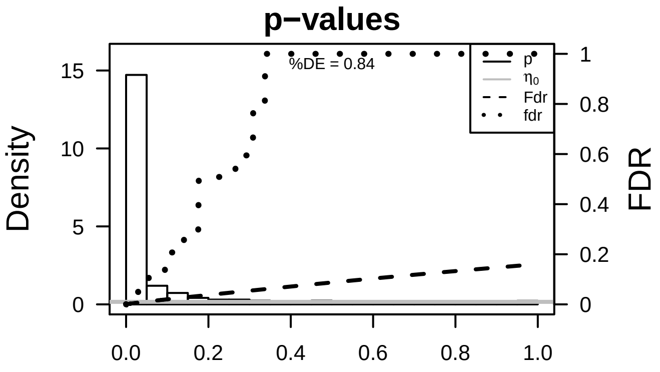

Supplement: Supplementary file 1 [file cells-09-00779-s001.zip › Supplementary materials/FileS4.zip!/FileS3/TFs - Results/Summary Sheets - Groups/Reports/Zhewan.1.10..pdf]
